# Starch accumulation in hulless barley during grain filling

**DOI:** 10.1186/s40529-017-0184-8

**Published:** 2017-07-14

**Authors:** Xu-guang Zheng, Jun-cang Qi, Hong-shan Hui, Li-hao Lin, Feng Wang

**Affiliations:** Department of Agronomy, College of Agriculture, Shihezi University, Shihezi, China

**Keywords:** Hulless barley (*Hordeum vulgare* L.), Starch accumulation, The ratio of amylopectin and amylose, Enzyme activity, Gene expression

## Abstract

**Background:**

Starch consists of two types of molecules: amylose and amylopectin. The objective of this study was increase understanding about mechanisms related to starch accumulation in hulless barley (*Hordeum vulgare* L.) grain by measuring temporal changes in (i) grain amylose and amylopectin content, (ii) starch synthase activity, and (iii) the relative expressions of key starch-related genes.

**Results:**

The amylopectin/amylose ratio gradually declined in both Beiqing 6 and Kunlun 12. In both cultivars, the activities of adenosine diphosphate glucose pyrophosphorylase, soluble starch synthase (SSS), granule bound starch synthase (GBSS), and starch branching enzyme (SBE) increased steadily during grain filling, reaching their maximums 20–25 days after anthesis. The activities of SSS and SBE were greater in Ganken 5 than in either Beiqing 6 or Kunlun 12. The expression of *GBSS I* was greater in Beiqing 6 and Kunlun 12 than in Ganken 5. In contrast, the expression of *SSS* *I*, *SSS* *II* and *SBE* *I* was greater in Ganken 5 than in Beiqing 6 and Kunlun 12. The peak in *GBSS* *I* expression was later than that of *SSS* *I*, *SSS* *II*, *SBE* *IIa* and *SBE* *IIb.* The *GBSS* *I* transcript in Kunlun 12 was expressed on average 90 times more than the *GBSS* *II* transcript.

**Conclusions:**

The results suggest that SBE and SSS may control starch synthesis at the transcriptional level, whereas *GBSS* *I* may control starch synthesis at the post transcriptional level. *GBSS* *I* is mainly responsible for amylose synthesis whereas *SSS* *I* and *SBE* *II* are mainly responsible for amylopectin synthesis in amyloplasts.

**Electronic supplementary material:**

The online version of this article (doi:10.1186/s40529-017-0184-8) contains supplementary material, which is available to authorized users.

## Introduction

Starch is the main end product of carbon fixation during photosynthesis. Starch consists of two major components, amylose and amylopectin. Amylopectin is composed of short α-1,4-linked chains of glucose. About 5% of these chains are linked together by α-1,6 linkages (Manners 1989; Buléona et al. 1998; Preiss and Sivak 1998). Amylose is also composed of glucose chains; however less than 1% of these chains are linked by α-1,6 branches (Imberty et al. 1991). The structure and relative proportion of amylose and amylopectin are the primary determinants of the physical and chemical properties of starch.

Several enzymes are involved in starch biosynthesis. ADP-glucose pyrophosphorylase (AGPP) catalyzes the first reaction in starch synthesis, producing the activated glucosyl donor ADP-glucose (James et al. 2003). Granule-bound starch synthase (GBSS) is involved in amylose synthesis. Soluble starch synthase (SSS) catalyses the elongation of amylopectin chains. Starch branching enzyme (SBE) introduces branch points into the amylopectin chains. The precise mechanism that controls starch biosynthesis in grain is complex and not well understood.

Amylopectin is formed by multiple isoforms of SSS (*SSS I*, *SSS II* and *SSS III*) and SBE (*SBE I*, *SBE IIa* and *SBE IIb*). Several multiple isoform enzymes are plant species-specific. Among the species examined so far, each isoform of SSS and SBE plays a distinct role in amylopectin biosynthesis (Nakamura 2002).

Recent studies suggest that cereal endosperms have distinct cytosolic and plastidial forms of AGPP which are encoded by separate large-subunit and small-subunit genes. The AGPP small subunit gene sequences from various eudicots and monocots differ primarily in exon (James et al. 2003). There are differing opinions about how *GBSS I* regulates starch synthesis. McCue et al. (2002) suggested that *GBSS I* controls starch synthesis in wheat at the transcriptional and post-transcriptional levels. Wang et al. (2014) studied starch biosynthesis in rice and proposed that *GBSS I* controls starch synthesis at the transcriptional level. There are few reports about the role of other enzyme genes on the synthesis and accumulation rate of amylose and amylopectin in grain. Information about the relative expression of genes encoding AGPP, GBSS, SSS and SBE at different development stages could provide insight about the mechanism controlling starch biosynthesis.

Hulless barley is mainly grown on the Tibetan Plateau. Hulless barley seeds consist primarily (63.2–65.3%) of starch. In fact, the process of grain filling in hulless barley is the process of starch accumulation. Hulless barley includes both waxy and non-waxy cultivars. The starch in waxy cultivars is composed of amylopectin (98.9%), whereas the starch in non-waxy cultivars is composed of both amylose (16.2–23.3%) and amylopectin (74.1–78.4%). The objectives of this experiment were (i) to compare the physiological and biochemical characteristics of starch biosynthesis during endosperm development in waxy and non-waxy cultivars of hulless barley and (ii) to study the differential expressions of AGPP, GBSS, SSS, and SBE genes at different grain filling stages.

## Materials and methods

### Plant materials and experiment design

The field experiment was conducted in 2015 at the Agriculture Experiment Station of Shihezi University, China. Three hulless barley cultivars were used in this study: Beiqing 6 (non-waxy), Kunlun 12 (non-waxy), and Ganken 5 (waxy).

The barley was sown in April. Each cultivar was grown in three replicate plots. The area of each plot was 5 m^2^. The row spacing was 20 cm. There was 2 cm between plants within each row. The fertilizer and irrigation practices were the same as those used by farmers in the regions.

Plants were tagged as they flowered. Forty spikes with the same flowering date were collected from each plot 5, 10, 15, 20, 25, 30, and 35 days after anthesis (DAA) (Additional file 1: Figure S1). The fresh weight of the spikes was determined. Several kernels (5 g) were removed from each spike and then immediately frozen in liquid N. The samples were stored at −70 °C for determination of enzyme activity. The remaining kernels were placed in paper bags, dried at 105 °C for 1 h, and then dried at 80 °C until constant weight. These samples were used for measuring starch accumulation.

### Starch analysis and calculations

**Fig. 1:**
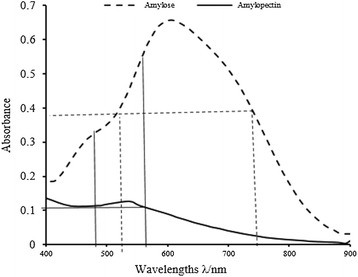
The main wavelength and the particular wavelength for amylose were 554 and 490 nm and the main wavelength and the particular wavelength for amylopectin were 542 and 713 nm

Starch, amylose, and amylopectin contents (mg grain^−1^) were determined according to the “double-wave-length” method of He (1981). In this method, standard solutions were prepared of amylose (5, 10, 15, 20, 25, and 30 μg mL^−1^) and amylopectin (40, 50, 60, 70, 80, 100, and 120 μg mL^−1^). The absorbance of the amylose standards was measured at 554 nm (A_λ1_) and 490 nm (A_λ2_) (Fig. 1). A standard curve was then prepared by plotting the amylose concentration on the x-axis and the average absorbance difference (ΔA, calculated by subtracting A_λ2_ from A_λ1_) on the y-axis. The linear regression equation of the standard curve was Y = 0.0079x − 0.0139 (R^2^ = 0.99). The standard curve for amylopectin was prepared in the same way only absorbance was measured at 542 nm (A_λ1_) and 713 nm (A_λ2_). The ΔA was calculated by subtracting A_λ2_ from A_λ1_. The linear regression equation of the standard curve was Y = 0.0059x + 0.0191 (R_2_ = 0.99). Total starch contents were determined by summing the amylose content and amylopectin content.

Next, after crushing to pass a 60 mesh sieve, the grain samples (0.1 g) were put into 100 mL beakers. Small amounts of anhydrous ethanol were added to the beakers followed by 10 mL of 1 mol L^−1^ NaOH. The beakers were then put into a water bath at 65 °C. After the starch had dissolved, the beakers were removed from the water bath and distilled water was added to bring the sample volume to 50 mL. The samples were then mixed and allowed to stand at room temperature.

A 3-mL aliquot of each solution was mixed with 20–30 mL distilled water. The pH of the solutions was adjusted to 3.5 with 0.1 mol L^−1^ HCl. A 0.5 mL aliquot of iodine reagent (0.1% I_2_ + 1% KI) was added to the samples and then distilled water was added to bring the final volume to 50 mL. The samples were mixed and allowed to stand at room temperature for 20 min. Standard solutions were prepared of amylose (0–30 μg mL^−1^) and amylopectin (40–120 μg mL^−1^).

The absorbance the main and particular wavelengths for amylose were 554 and 490 nm, respectively. The main and particular wavelengths for amylopectin were 542 and 713 nm, respectively (Fig. 1). Total starch content (%) was calculated as the sum of the amylose content (%) and the amylopectin content (%). Starch accumulation (mg grain^−1^) was calculated by multiplying the starch content (%) by the seed weight (mg grain^−1^).

The absorbance of the sample solution at λ1, λ2, λ3 and λ4 was determined by spectrophotometer. The straight and amylopectin content of the samples was calculated according to the double wavelength standard curve of straight and amylopectin. The sum of the two samples was determined by the total starch content.

$${\text{w}}1 = \frac{{\left( {A554 \, {\text{nm}} - A490 \, {\text{nm}}} \right) + 0.0139}}{0.079*M*10} ; {\text{w}}2 = \frac{{\left( {A542 \, {\text{nm}} - A713 \, {\text{nm}}} \right) - 0.0191}}{0.059*M*10};w = w1 + w2$$

Note: W is the total starch content/%; W1 is the amylose content/%; W2 is the amylopectin content/%; M is the sample mass/g; 10 is the unit conversion factor.

The grain starch accumulation process can be described by the logistic equation W = K/(1 + ae^−bt^), where *W* is starch accumulation, *t* is days after anthesis, *K* is the maximum accumulation amount, and *a* and *b* are constants. The equation can be rewritten as: y = k/(1 + e^A^ + BT) where A = ln (a) and B = −b. The following characteristics were calculated for starch, amylopectin and amylose accumulation using CurveExpert 1.4 software: (i) time when the starch accumulation rate was at its maximum (Tmax) = −A/B; (ii) starch accumulation at 90% of its final amount (D) = [ln (1/9) − A]/B; (iii) the maximum starch accumulation rate (V_max_) = −KB/4; (iv) the mean starch accumulation rate (V_mean_) = K/D; and (v) the initial growth power (C_0_) = K/(1 + e^A^) (Wang et al. 2014).

### Enzyme isolation and assays

Enzyme extraction was done using the method of Cheng and Jiang (2001). Twenty fresh kernels were placed into 5 mL buffer solution (50 mmol L^−1^ Hepes–NaOH, pH 7.5; 8 mmol L^−1^ MgCl_2_, 2 mmol L^−1^ EDTA_2_, 10 g L^−1^ PVP-30 and 1 mmol L^−1^ DTT). The samples were homogenized by grinding in an ice bath and then centrifuged at 10,000 rpm for 30 min at 4 °C. The supernatant, referred to as enzyme preparation 1, was used to determine the activities of AGPP, SSS, and SBE activity. The sediment was resuspended with 5 mL of buffer solution and then centrifuged at 1000 rpm for 3 min at 4 °C. The supernatant, referred to as enzyme preparation two, was used to determine GBSS activity.

The AGPP activity was measured by mixing enzyme preparation 1 (20 μL) into a reaction mixture(110 μL) consisting of 100 mmol L^−1^ Hepes–NaOH (pH 7.4), 1.2 mmol L^−1^ ADPG, 3 mmol L^−1^ PPi, 5 mmol L^−1^ MgCl_2_ and 4 mmol L^−1^ DTT. The total reaction volume was 130 μL. The solutions were kept at 30 °C for 20 min and then the reactions were terminated by placing the samples in boiling water for 1 min. The solutions were centrifuged at 10,000 rpm for 10 min. A 100-μL aliquot of the supernatant was collected and mixed with 5.2 μL of colorizing agent consisting of 5.76 mmol/L NADP, 0.08 unit P-gucomutase, and 0.07 unit G6P-dehydrogenase. The absorption of the samples was measured at 340 nm. One unit of AGPP activity was defined as the amount of enzyme causing a one unit per min increase in absorbance.

The GBSS activity was measured by mixing enzyme preparation 1 (20 μL) into a reaction mixture (36 μL) consisting of 50 mmol L^−1^ Hepes–NaOH (pH 7.4), 1.6 mmol L^−1^ ADPG, 0.7 mg amylopectin, and 15 mmol L^−1^ DTT. The total reaction volume was 56 μL. The solutions were kept at 30 °C for 20 min. The reactions were terminated by placing the reaction mixtures in boiling water for 1 min followed by cooling in a water bath. Next, the samples were mixed with 20 μL of a reaction solution consisting of 50 mmol L^−1^ Hepes–NaOH (pH 7.4), 4 mmol L^−1^ PEP, 200 mmol L^−1^ KCl, 10 mmol L^−1^ MgCl_2_, and 1.2 units of pyruvate kinase. The solutions were kept at 30 °C for 20 min. The reactions were terminated by placing the mixture in boiling water for 1 min. The samples was centrifuged at 10,000 rpm for 10 min. A 60-μL aliquot of the supernatant was collected and then mixed with 43 μL of color liquid including 50 mmol L^−1^ Hepes–NaOH (pH 7.4), 10 mmol L^−1^ glucose, 20 mM MgCl_2_, 2 mmol/L NADP, 1.4 units hexokinase, and 0.35 units G-6-P-dehydrogenase. The solutions were kept at room temperature for 10 min. The enzyme activity was assayed spectrophotometrically at 340 nm. One unit of GBSS activity was defined as the amount of enzyme causing a one unit per min increase in absorbance (Nakamura et al. 1989).

The SBE activity was measured by mixing 50 μL of enzyme preparation 1 into a reaction mixture containing 50 mM Hepes–NaOH (pH 7.4), 5 mM G1P, 1.25 Mm AMP, and phosphorylase a (54 unit). The total reaction volume was 1.45 mL. The solutions were kept at 30 °C for 20 min and then the reactions were terminated by placing the samples in boiling water for 1 min. The reaction was terminated by adding 50 μL of 1 mol L^−1^ HCl. The solutions were then mixed with 500 μL dimethylsulfoxide and 700 μL of iodine reagent (0.1% I_2_ + 1% KI). The enzyme activity was assayed spectrophotometrically at 540 nm. One unit of SBE activity was defined as the amount of enzyme causing a one unit per min increase in absorbance.

The procedure for measuring SSS activity used enzyme preparation 2 rather than enzyme preparation 1. In all other aspects, the procedure to determine SSS activity was the same as that used to determine GBSS activity. One unit of GBSS activity was defined as the amount of enzyme causing a one unit per min increase in absorbance at 340 nm.

### Gene expression and calculation of the expression rate

Gene expression was performed using a reverse transcriptase polymerase chain reaction (RT-PCR). Total RNA from endosperm was isolated using Fruit-mate™ for RNA purification (TaKaRa) and RNAiso Plus (TaKaRa Code: 9108). The RNA was reverse transcribed with the Script cDNA synthesis kit (TaKaRa). The synthesized cDNA was subjected to PCR for 45 cycles. The conserved regions of the gene sequences of *AGPP* (Tan et al. 2010), *GBSS* *I*, *GBSS* *II*, *SSS I*, *SSS II*, *SSIII*, *SBE I*, *SBE IIa*, *SBE IIb* (Wang et al. 2014) were obtained from wheat and used to design primers for detecting gene expression in wheat endosperm. The wheat actin gene (NCBI Accession No. DN551593) was used as a reference control. The fragment size of the sequences, annealing temperature (Tm), and real-time PCR efficiency are listed in Table 1. Aliquots of the individual PCR products were resolved by agarose gel electrophoresis and the bands were visualized with a PhotoDoc-It imaging system (UVP, LLC, Upland, CA, USA).

**Table 1:** Primer sequence for starch synthesis related gene

Gene	NCBI accession	Forward primers (5′–3′)	Reverse primers (3′–5′)	Annealing temperature (°C)
AGPP	AK359692	TTCAGTTTCATGACCGTTC	AGTATCCATCTGTCTCCCTC	60
*GBSS I*	AY050174	GACACTATCGTGGAAGGCAAG	TTGACCATCTCATGGTACGC	60
*GBSS II*	AF109395	CACAGAATGCCAGAGGCATAG	GAACAGATGGGAATCACTCCA	60
*SSS I*	AJ292521	GCAAAAGGAGAGGAGGGTACA	ACGTATGGTCTTTCGTCATGC	60
*SSS II*	AB201445	GCTACACCAACTTCTCCCTG	GATGATCTCCACGCCCTTCT	60
*SSS III*	AF258608	GACATGTGGTTTTGCTTGGTT	AGCCAGCGTATATCAGGTGAG	60
*SBE I*	AF286317	ATGTTTGGTGGACATGGAAGA	ATGTTTGGTGGACATGGAAGA	60
*SBE IIa*	Y11282	ATGTTTGGTGGACATGGAAGA	ATGTTTGGTGGACATGGAAGA	60
*SBE IIb*	AY740401	ATGTTTGGTGGACATGGAAGA	ATGTTTGGTGGACATGGAAGA	60
Actin	DN551593	GGAAAAGTGCAGAGAGACACG	TACAGTGTCTGGATCGGTGGT	60

The RT-PCR efficiencies (E) were calculated from the slopes in Light Cycler software using the equation E = 10[−1/slope]. Calculations for the test precision and test variability are based on the crossing-point (CP) variation from the CP mean value. A mathematical model was used to determine the relative expression ratio (R) of an unknown sample to the control, and expressed in comparison to a reference gene (Wang et al. 2014).

$${\text{Ratio}} = \left( {{\text{E}}_{\text{target}} } \right)^{{\vartriangle {\text{CP}}\left( {{\text{control}} - {\text{sample}}} \right)}} /\left( {{\text{E}}_{\text{ref}} } \right)^{{\vartriangle {\text{CP}}\left( {{\text{control}} - {\text{sample}}} \right)}} .$$

Actin gene was used as a reference control because it has highly conserved proteins that are involved in cell motility, structure and integrity.

### Statistical analysis

The data were analyzed with SPSS 19.0 and Excel 2013 software. Samples were analyzed in triplicate and all data are presented as means. Logistic equations were fit with CurveExpert v. 1.4 software to describe the relationship between starch accumulation quantity (y) and days after flowering (x).

## Results

### Starch accumulation

**Fig. 2:**
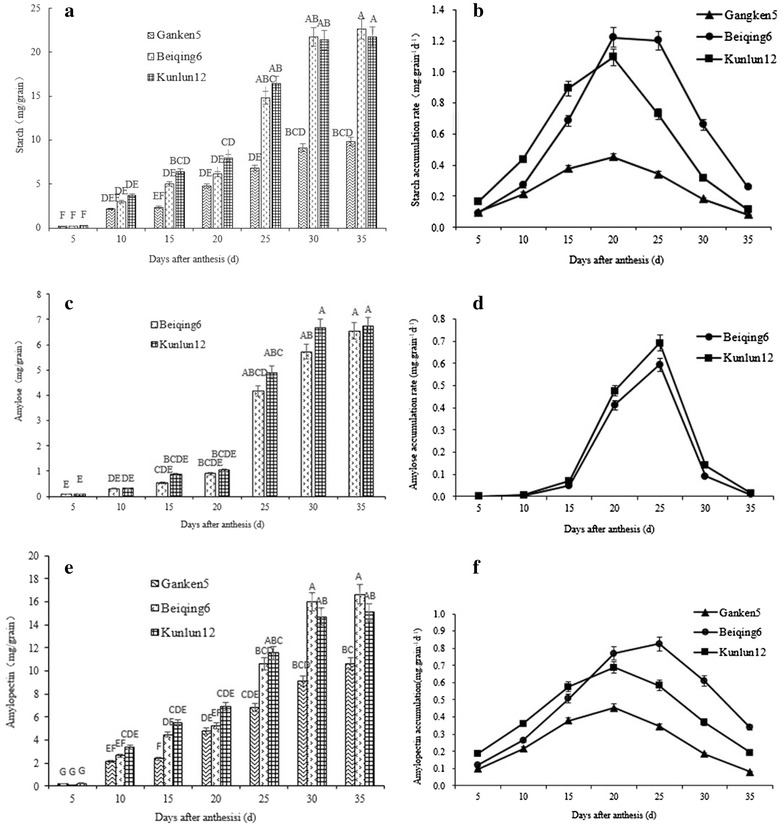
Temporal changes in the content and accumulation rate in three hulless barley cultivars.** a** Starch content; **b** Starch accumulation rate; **c** Amylose content; **d** Amylose accumulation rate; **e** Amylopectin content; **f** Amylopectin accumulation rate. *Bars* with* different letters* are significantly different at the 0.01 level of significance. *Error bars* indicate standard deviation

Starch, amylose, and amylopectin contents increased across time in all three cultivars (Fig. 2a, c, e). Starch accumulation at flowering was less in Ganken 5 than in either Beiqing 6 or Kunlun 12. The starch content was less in Beiqing 6 than in Kunlun 12 at 10, 15, 20, and 25 DAA; however the difference between the two cultivars became less with time. The starch content of Beiqing 6 at 30 and 35 DAA was 1.62 and 3.91% greater, respectively, compared with Kunlun 12. The temporal changes in amylopectin content were similar to those of starch. The amylopectin contents of Beiqing 6 at 30 and 35 DAA were 8.12 and 9.43% greater, respectively, than of Kunlun 12. The amylose content was greater in Kunlun 12 than in Beiqing 6 from 5DAA to 35DAA during grain filling. The amylose content of Ganken 5 was too low to be detected.

Grain starch contents are affected by the starch accumulation rate during grain filling. There were temporal differences in the accumulation rates of starch, amylose, and amylopectin among the three hulless barley cultivars (Fig. 2b, d, f). These differences became significant 15–25 DAA. The accumulation rates of starch and amylopectin decreased in the order Kunlun 12 > Beiqing 6 > Ganken 5 until 18 DAA. During the remainder of grain filling, the accumulation rates decreased in the order Beiqing 6 > Kunlun 12 > Ganken 5. The temporal changes in starch and amylopectin accumulation rate were consistent with the changes in starch and amylopectin content. The amylose accumulation rate was greater in Kunlun 12 than in Beiqing 6 during grain filling and, as a result, Kunlun 12 had the highest final amylose content (7.21 mg grain^−1^).

The data in Fig. 2a, c, e were fit with logistic equations to describe the temporal changes in grain starch content. The equations and their parameters are shown in Table 2. For starch, the maximum accumulation rate, the average accumulation rate, and the initial growth power were all less in Ganken 5 than in Beiqing 6 and Kunlun 12. The initial growth power of Ganken 5 was less than that of the other cultivars because (i) the starch in Ganken 5 is mainly composed of amylopectin and (ii) amylopectin synthesis is more complex than amylose synthesis. The maximum starch accumulation rate, the time to the maximum accumulation rate, the accumulation duration, the average accumulation rate, and the initial growth power were greater in Kunlun 12 than in Beiqing 6. Thus, the final starch content of non-waxy hulless barley depends on both the amylose accumulation rate and the initial growth potential. For amylopectin, the maximum accumulation rate, the time to reach the maximum accumulation rate, the accumulation duration, the average accumulation rate, and the initial growth power were greater in Ganken 5 than in Beiqing 6 and Kunlun 12. This indicated that the final amylopectin content depends on the maximum amylopectin accumulation rate and on the average accumulation rate. The onset and termination times of amylopectin accumulation did not affect the final amylopectin content.

**Table 2:** Logistic equations describing grain starch accumulation in three hulless barley cultivars

Cultivars	Poly-saccharide	Logistic equation	Correlation coefficient	T_max_/d	D/d	V_mean_/(mg grain^−1^ d^−1^)	V_max_/(mg grain^−1^ d^−1^)	C_0_
Ganken 5	Starch	y = 9.358/(1 + e^3.780 − 0.194X^)	0.982**	19.46	30.78	0.304	0.454	0.209
	Amylose	–	–	–	–	–	–	–
	Amylopectin	y = 9.358/(1 + e^3.779 − 0.194X^)	0.982**	19.46	30.79	0.304	0.454	0.209
Beiqing 6	Starch	y = 23.839/(1 + e^5.510 − 0.222X^)	0.982**	22.84	32.76	0.728	1.32	0.15
	Amylose	y = 5.834/(1 + e^11.522 − 0.497X^)	0.990**	23.17	27.59	0.212	0.725	0.0001
	Amylopectin	y = 19.008/(1 + e^4.139 − 0.177X^)	0.981**	23.39	35.80	0.531	0.841	0.298
Kunlun 12	Starch	y = 19.432/(1 + e^4.352 − 0.227X^)	0.978**	19.13	28.79	0.675	1.105	0.247
	Amylose	y = 7.046/(1 + e^10.573 − 0.454X^)	0.982**	23.31	28.16	0.25	0.799	0.0002
	Amylopectin	y = 16.389/(1 + e^3.380 − 0.168X^)	0.977**	20.10	33.18	0.494	0.689	0.54

y, grain starch accumulation; x, days after anthesis

*T*_*max*_ time to reach the maximum accumulation rate, *D* accumulation duration, *V*_*mean*_ mean accumulation rate, *V*_*max*_ maximum accumulation rate, *C*_*0*_ initial growth potential

** Significant at the 0.01 probability level

**Fig. 3:**
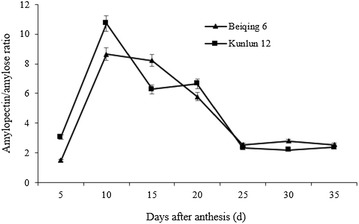
The ratio of amylopectin and amylose in Beiqing 6 and Ganken 5. *Error bars* indicate standard deviation

The amylopectin/amylose ratio increased to a maximum and then decreased in both Beiqing 6 and Kunlun 12 (Fig. 3). This shows that the amylopectin synthesis rate was greater than the amylose synthesis rate between 5 and 10 DAA, whereas the opposite was true between 10 and 25 DAA. The amylopectin/amylose ratio at 10 DAA was 10.72 for Kunlun 12 and 8.64 for Beiqing 6. The ratio slowly declined after 15 DAA. There was no significant difference in the amylopectin/amylose ratio of Kunlun 12 and Beiqing 6 between 15 and 30 DAA.

### Starch synthesis enzymes

#### Adenosine diphosphate glucose pyrophosphorylase (AGPP) activity

**Fig. 4:**
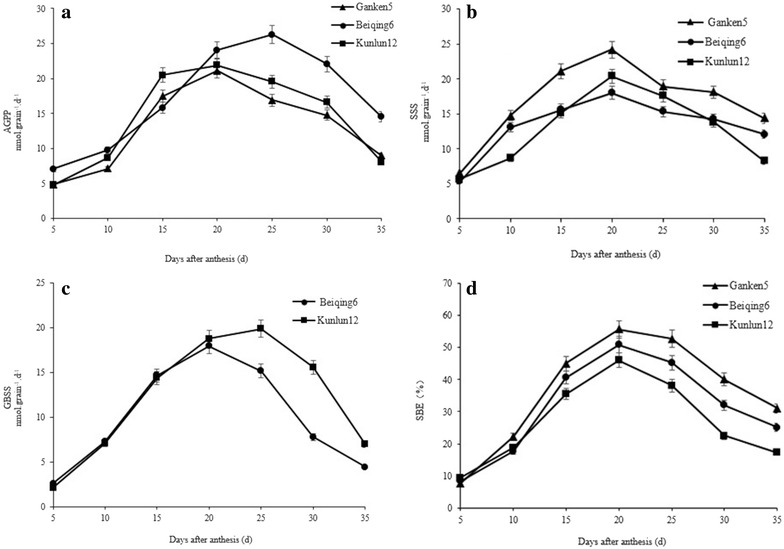
Activity of AGPP, SSS, GBSS and SBE in three hulless barley cultivars during grain filling. **a** AGPP enzyme; **b** SSS enzyme; **c** GBSS enzyme; **d** SBE enzyme. *Error bars* indicate standard deviation

The AGPP activity increased to a maximum and then decreased in all three cultivars (Fig. 4a). There were differences among cultivars both in the maximum activity and in the time of maximum activity. Beiqing 6 had the highest maximum AGPP activity, followed by Kunlun 12 and then Ganken 5. The enzyme activity of AGPP of Beiqing 6 reached 26.28 nmol grain^−1^ d^−1^ at 25 DAA, whereas Kunlun 12 reached 21.86 nmol grain^−1^ d^−1^ and Ganken 5 reached 21.10 nmol grain^−1^ d^−1^ at 20 DAA.

#### Soluble starch synthase (SSS) activity

The SSS activity in all three cultivars increased to a maximum about 20 DAA and then decreased (Fig. 4b). There were significant differences in SSS activity among the cultivars. During grain filling, SSS activity changed less in Beiqing 6 than in either Ganken 5 or Kunlun 12. The SSS is mainly involved in amylopectin synthesis. At 20 DAA, Ganken 5 had the highest SSS activity (24.21 nmol grain^−1^ d^−1^) followed by Kunlun 12 (20.39 nmol grain^−1^ d^−1^) and then Beiqing 6 (18.00 nmol grain^−1^ d^−1^). This is because amylopectin is the major component of starch in Ganken 5. Overall, SSS activity was greater in Ganken 5 than in either Kunlun 12 or Beiqing 6 during the entire grain filling process.

#### Granule-bound starch synthase (GBSS) activity

No GBSS activity was detected in Ganken 5 during grain filling (Fig. 4c). There was no significant difference in GBSS activity between Kunlun 12 and Beiqing 6 during early grain filling. However, during late grain filling, GBSS activity was significantly greater in Kunlun 12 than in Beiqing 6. The maximum GBSS activities were observed 20 DAA in Beiqing 6 (17.95 nmol grain^−1^ d^−1^) and 25 DAA in Kunlun 12 (19.87 nmol grain^−1^ d^−1^). The amylose content of Kunlun 12 was also greater than that of Beiqing 6. Overall, these results indicated that GBSS regulates amylose synthesis in barley and determines the amylose content of the grain. The role of GBSS is more important during late grain filling than during early grain filling.

#### Starch branching enzyme activity

The SBE directly affects grain amylopectin content. The SBE can cut α-1, 6-glycosidic bonds, thereby forming branching sugar chains. The SBE activity in all three cultivars increased to a maximum about 20 DAA and then declined (Fig. 4d). Ganken 5 had the highest maximum SBE activity, followed by Beiqing 6 and then Kunlun 12. These results indicated that the waxy cultivar, Ganken 5, had more ability to synthesize amylopectin than the non-waxy cultivars, Beiqing 6 and Kunlun 12.

#### Correlation between starch synthase activity and starch accumulation rate

Previous studies indicated that starch accumulation rates were more consistently related to the activity of AGPP than to the activities of SSS, GBSS, and SBE (Liu et al. 2004). However, in the present study, the activities of AGPP, SSS, GBSS, and SBE were all significantly or highly significantly correlated with starch accumulation. This indicates that these enzymes play an important regulatory role in starch synthesis in hulless barley. The activities of AGPP, GBSS, and SBE were significantly positively correlated with amylose accumulation rate. The correlation between SSS activity and amylose accumulation rate was not significant, probably because SSS is mainly involved in amylopectin synthesis (Table 3).

**Table 3:** Correlation analysis between enzyme activity and starch accumulation rate in three hulless barley cultivars at grain filling

Enzyme	Cultivar	Amylose accumulation rate	Amylopectin accumulation rate	Starch accumulation rate
AGPP	Ganken 5	–	0.878**	0.878**
	Beiqing 6	0.829*	0.983**	0.925**
	Kunlun 12	0.668	0.934**	0.871*
SSS	Ganken 5	–	0.857*	0.857*
	Beiqing 6	0.556	0.848*	0.838*
	Kunlun 12	0.57	0.886**	0.841*
GBSS	Ganken 5	–	0.952**	0.592**
	Beiqing 6	0.748*	0.854*	0.924**
	Kunlun 12	0.751*	0.993**	0.970**
SBE	Ganken 5	–	–	–
	Beiqing 6	0.765*	0.878**	0.935**
	Kunlun 12	0.760*	0.966**	0.944**

* and ** indicate significance at the 0.05 and 0.01 probability levels, respectively

#### Gene expression

**Fig. 5:**
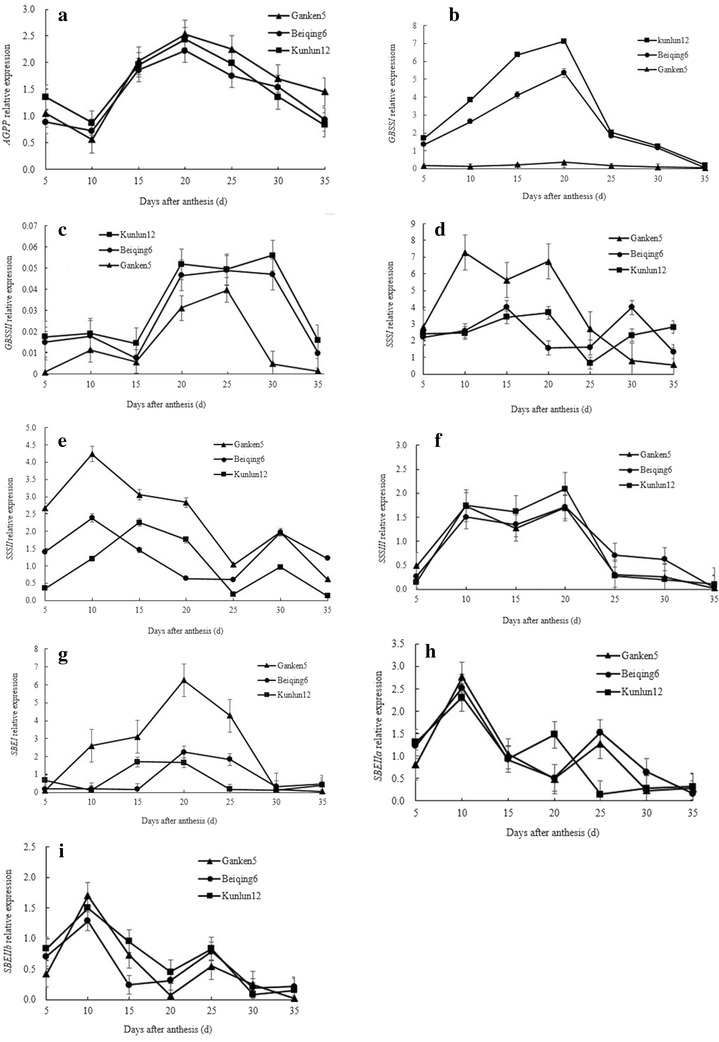
Changes in enzyme gene expression during grain filling. **a** *AGPP* relative expression; **b** *GBSS I* relative expression; **c** *GBSS II* relative expression; **d** *SSS I* relative expression; **e** *SSS II* relative expression; **f** *SSS III *relative expression;** g** *SBE I *relative expression; **h** *SBE IIa *relative expression;** i** *SBE IIb * relative expression.* Error bars* indicate standard deviation

AGPP catalyzes the first reaction in starch synthesis, producing the activated glucosyl donor ADP-glucose (ADPG). AGPP is comprised of two large subunits and two small subunits, each of which is encoded by distinct genes. Our study indicated no significant differences in *AGPP* expression between the waxy hulless barely, Ganken 5, and the non-waxy hulless barely, Beiqing 6 and Ganken 12 (Fig. 5a).

GBSS consists of two forms: *GBSS I* and *GBSS II*. The expression of *GBSS* *I* appears to be mostly confined to storage tissues. *GBSS* *II* is encoded by a separate gene and is generally expressed more in leaves and other non-storage tissues that accumulate transient starch. The results of the present study indicated that the relative expression of *GBSS* *I* increased to a maximum at 20 DAA and then declined in the non-waxy cultivars Beiqing 6 and Kunlun 12 (Fig. 5b). In contrast, the relative expression of the waxy cultivar Ganken 5 was much less and did not change with time. The relative expression of *GBSS* *II* increased with time in all three cultivars, reaching maximums between 20 and 30 DAA (Fig. 5c). The relative expression of *GBSS I* was on average 90 times greater than that of *GBSS* *II* in Beiqing 6 and Kunlun 12 during grain filling. Furthermore, the relative expression of *GBSS* *II* was much less than that of the other starch synthesis genes. Overall, low *GBSS* *I* expression in Ganken 5 combined with the cultivar’s low amylose concentrations suggests that *GBSS* *I* is mainly responsible for amylose synthesis in cereal endosperm. Although its relative expression is low in endosperm, *GBSS* *II* may be essential to starch synthesis in other non-storage tissues. Low *GBSS* *I* expression in Ganken 5 indicates why amylose concentrations are non-detectable in waxy Ganken 5.

The SSS genes are exclusively involved in amylopectin biosynthesis. The distribution of the SSS genes between the stroma and starch granules within plastids varies among plant species, tissue types, and plant development stages. In our study, the relative expression of *SSS* *I* and *SSS* *II* was significantly greater in Ganken 5 than in Beiqing 6 and Kunlun 12 between 5 and 25 DAA (Fig. 5d, e). The relative expression of *SSS* *I*, *SSS* *II*, and *SSS* *III* exhibited similar patterns across time in Beiqing 6 and Kunlun 12 (Fig. 5d–f). The relative expression of *SSS I* in Ganken 5 was already high at the earliest phase of seed formation (5 DAA). The *SSS I* expression increased to peak at 10 DAA, but then declined. The *SSS I* expression of Ganken 5 was significantly less than that of Beiqing 6 and Kunlun 12 between 25 and 35 DAA. The SSS genes are exclusively involved in amylopectin biosynthesis and *SSS I* is the major SSS form in cereal endosperm. This may be one reason for the decline in the amylopectin/amylose ratio in Beiqing 6 and Kunlun 12.

The SBE gene can combine α-1,6 linkages by cleaving internal α-1,4 bonds and then transferring the released reducing ends to C6 hydroxyls. Two SBE gene classes (i.e., *SBE* *I* and *SBE* *II*) differ in the length of glucan chains transferred in vitro and in their substrate specificities. In cereals, there are two closely related forms of *SBE* *II* (i.e., *SBE* *IIa* and *SBE* *IIb*). These two forms differ in chain-length specificity in vitro, with *SBE* *IIb* transferring shorter chains than *SBE* *IIa* during extended incubation. In the present study, temporal changes in *SBE* *I*, *SBE* *IIa*, and *SBE* *IIb* were similar in all three cultivars (Fig. 5h, i, j). However, the relative expression of *SBE* *I* was significantly greater in waxy Ganken 5 than in Beiqing 6 and Ganken 5. This suggested that *SBE* *I* has a major influence on SBE activity and on amylopectin accumulation during endosperm development.

## Discussion

Starch biosynthesis in grain has been studied extensively in wheat and rice but not in hulless barely (Li and Sun 1980; Nakamura and Yuki 1992). We observed that starch and its components increased with time during grain filling. The patterns were similar to those observed in wheat and rice (Li and Sun 1980; Jian et al. 2002). The starch and amylose accumulation rates were always greater in the non-waxy cultivars (i.e., Beiqing 6 and Kunlun 12) than in the waxy cultivar (Ganken 5). The amylopectin content of non-waxy Beiqing 6 was significantly greater than that of waxy Ganken 5 but not greater than that of non-waxy Kunlun 12.

The roles of AGPP, SSS, GBSS, and SBE in starch biosynthesis have been investigated in previous studies (Li and Sun 1980; Doehlert et al. 1988; Nakamura and Yuki 1992). However, the contribution of each enzyme to starch synthesis is still disputed. Our results showed that the maximum activities of AGPP in Beiqing 6 was 26.28 nmol grain^−1^ d^−1^ and in Kunlun 12 was 21.86 nmol grain^−1^ d^−1^ were greater in Ganken 5 was 21.10 nmol grain^−1^ d^−1^. Furthermore, the activities of AGPP, SSS, GBSS, and SBE were significantly positively correlated with the starch accumulation rate during grain filling. This indicated that AGPP, SSS, and GBSS play important roles in starch biosynthesis in hulless barley. Preiss et al. (1988) reported that AGPP was the rate-limiting enzyme in starch biosynthesis and that AGPP activity was related to the synthesis rate and final starch amount. Okita (1992) observed that SSS activity was consistent with starch accumulation rate. We observed peaks in the activities of AGPP, SSS, and GBSS between 20 and 25 DAA in all three cultivars. This was also the time when starch accumulation rates were highest.

AGP is composed of two large subunits and two small subunits, each of which is encoded by distinct genes (*AGPP*-*L* and *AGPP*-*S*). The enzyme is known to be largely extraplastidial (i.e., 85–95% cytosolic) in cereal endosperm, but plastidial in other cereal tissues and in all tissues of non-cereal plants (Fig. 5a) (Beckles et al. 2001). AGPP catalyzes the first reaction in starch synthesis, producing the activated glucosyl donor ADP-glucose. Cao (2012) observed that waxy and non-waxy wheat differed significantly both in *AGPP*-*L* expression and in *AGPP*-*S* expression. However, in this study, AGPP activity and the relative expression of AGPP exhibited similar temporal changes in all three cultivars. This may be because AGPP expression was different in wheat than in hulless barley. Additional research needs to be done to confirm this.

Studies of waxy mutations in wheat and other cereals have shown that null mutations in genes encoding granule-bound starch synthase I (*GBSS I*) result in amylose-free starch in endosperm and pollen grains. The waxy locus in cereals is encoded by *GBSS* *I*, which catalyzes the elongation of amylose. In addition to its role in amylose biosynthesis, *GBSS* *I* is also responsible for the extension of long glucans within the amylopectin fraction (van de Wal et al. 1998). In the present study, GBSS enzyme activity peaked later than SBE and SSS (Fig. 5b, d, i, j). This suggested that SBE and SSS may control starch synthesis at the transcriptional level, and that *GBSS* *I* may control starch synthesis at the post transcriptional level.

Biochemical evidence suggests that *SSS* *I* is primarily responsible for the synthesis of the shortest glucan chains with a polymerization degree of 10 glucosyl units or less (Li et al. 2000). Further extension of the chains is achieved by the activities of the *SS*-*II* and *SS*-*III* isoforms (Li et al. 2000). In the present study, SSS genes were exclusively involved in amylopectin biosynthesis in hulless barley endosperm. Furthermore,* SSS I* was the major SSS form. The relative expression of *SSS* *I* decreased steadily across time (Fig. 5d), and there was a corresponding decrease in the amylopectin synthesis rate (Fig. 2f). This may be one reason for the decline in the amylopectin/amylose ratio across time (Fig. 3).

Two SBE gene classes (*SBE* *I* and *SBE* *II*) differ in the length of glucan chains transferred in vitro and in their substrate specificities. *SBE* *II*, compared with *SBE* *I*, transfers shorter chains, exhibits greater affinity towards amylopectin, and exhibits greater rates of branching with amylose (Guan and Preiss 1993). The results of the present study indicated similar expression profiles for *SBE* *IIa* and *SBE* *IIb* in endosperm. Both *SBE* *II* genes were expressed at high levels from 5 DAA to 10 DAA, but were then down-regulated after 15 DAA (Fig. 5i, j). Declines in the expression of the *SBE* *II* genes may be another reason for the decline in the amylopectin/amylose ratio across time (Fig. 3). Overall, the results suggested that *SBE* *II* plays an important role during early stages of endosperm development in hulless barley.

## Conclusion

Overall, the results indicated that the activities of AGPP, SSS, GBSS and SBE had some correlation with the rates of starch synthesis during grain filling in both the waxy and non-waxy cultivars. GBSS has an important effect on amylose synthesis, especially during late grain filling. SSS and SBE are associated with amylopectin biosynthesis. It remains unclear how AGPP, SSS, GBSS and SBE activities are coordinated to control starch biosynthesis rates. Further research will aim to identify direct interactions between starch biosynthetic enzymes, as well as the factors that regulate the enzyme activities.

Transcriptome analysis has the advantage of being able to quantify changes in gene transcript levels at different developmental stages in wild-type species, their mutants, and cultivars with different genetic backgrounds. Therefore, expression profiling could (i) lay a foundation for identifying genes involved in the regulation of starch metabolism and (ii) provide valuable insight into the mechanism of metabolic regulation of starch biosynthesis under various physiological conditions.

The amylopectin/amylose ratio gradually declined in both Beiqing 6 and Kunlun 12 (Fig. 4). Furthermore, the peak in *GBSS I* expression was later than that of *SSS* *I*, *SSS* *II*, *SBE* *IIa,* and *SBE* *IIb*. This may explain the decline in the ratio of amylopectin to amylose during grain filling in Beiqing 6 and Kunlun 12 (Additional file 1: Figure S1). The grain of Ganken 5 has high amylopectin content but almost no amylose. *GBSS I* expression was less in Ganken 5 than in Beiqing 6 and Kunlun 12. Overall, we conclude that *GBSS I* is mainly responsible for amylose synthesis whereas *SSS I* and *SBE II* are mainly responsible for amylopectin synthesis in amyloplasts.

## Additional file

**Additional file 1: Figure S1.** Morphology of different grains in different periods.

## Abbreviations

AGPP: adenosine diphosphate glucose pyrophosphorylase
SSS: soluble starch synthase
GBSS: granule bound starch synthase
SBE: starch branching enzyme
DAA: days after anthesis
