# Supplementary figures and images for: Starch accumulation in hulless barley during grain filling
Source: Bot Stud. 2017 Jul 14;58:30. doi: 10.1186/s40529-017-0184-8 (PMC5511127; doi:10.1186/s40529-017-0184-8)

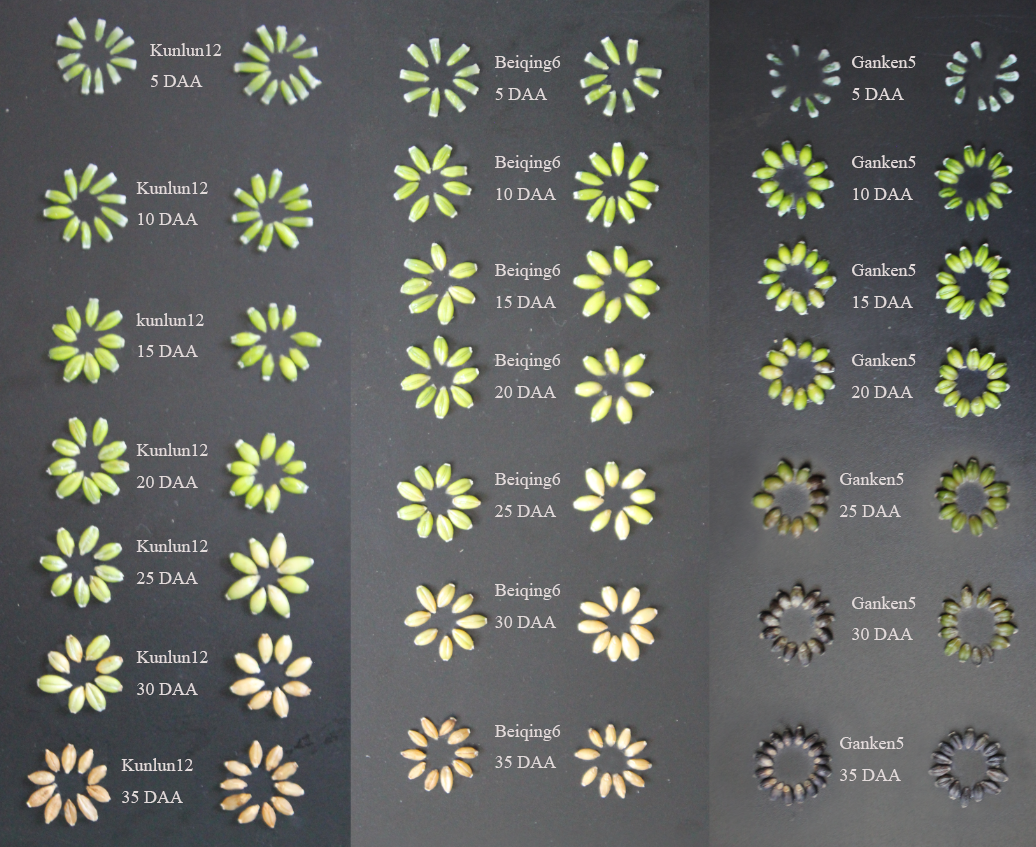

Supplement: Supplementary file 1 — Additional file 1: Figure S1. Morphology of different grains in different periods. [file 40529_2017_184_MOESM1_ESM.tif]
